# Endovascular embolization of post-tonsillectomy pseudoaneurysm in adults

**DOI:** 10.1186/s42155-025-00539-w

**Published:** 2025-04-01

**Authors:** Xiaodong Yang, Jiani Zhao, Rongrong Quan, Qiang Liu

**Affiliations:** Department of Radiology, The Second Affiliated Hospital of Xi’an Jiaotong University, Shaanxi Province, Xi’an, China

**Keywords:** Post-tonsillectomy hemorrhage, Pseudoaneurysm, Endovascular embolization, NBCA

## Abstract

**Objective:**

This study aims to evaluate the efficacy and safety of endovascular embolization in the treatment of post-tonsillectomy pseudoaneurysms.

**Methods:**

We conducted a retrospective analysis of four consecutive adults who experienced secondary post-tonsillectomy hemorrhage (PTH) due to pseudoaneurysms. Hemoglobin loss was identified to access the blood loss of patients. All patients underwent endovascular embolization of the injured artery using superselective catheterization techniques.

**Results:**

The angiogram revealed pseudoaneurysms in the ascending palatine artery in two patients, the facial artery in one patient, and the lingual artery in the other patient. Two patients were treated with endovascular embolization using n-butyl-2-cyanoacrylate (NBCA) glue, one patient was treated with coils, and one received a combination of coils and NBCA glue for embolization. All procedures were successful, with no clinical complications or rehemorrhage reported.

**Conclusion:**

Endovascular embolization is an effective, feasible, and safe treatment option for the patients with post-tonsillectomy pseudoaneurysm. NBCA glue can be an effective and appropriate embolic material, but attention must be paid to the critical techniques involved.

## Introduction

Tonsillectomy is the most widely performed procedure in otolaryngology. Achieving complete and adequate hemostasis post-tonsillectomy is considered challenging due to the rich arterial blood supply of the tonsils. Hemorrhage is the most prevalent post-tonsillectomy complication, occurring in approximately 3–4% of cases [1]. Primary post-tonsillectomy hemorrhage (PTH), occurring within the first 24 h post-surgery, is generally attributed to the surgical technique or underlying coagulopathy. Conversely, secondary PTH, typically arising 5–10 days postoperatively due to the sloughing of the pseudomembrane from the tonsillectomy wound, is often associated with pseudoaneurysm formation [2, 3]. Pseudoaneurysm can lead to excessive and uncontrollable hemorrhage, which is a leading cause of mortality in patients following tonsillectomy [4–6].

Studies indicate that adults have a threefold higher likelihood of requiring surgical intervention for PTH compared to children, with a lower spontaneous resolution rate and more severe bleeding [7–9]. Traditional surgical approaches for managing PTH have involved local measures and ligation of the external carotid artery; however, these methods can sometimes prove ineffective [1, 10]. In recent years, endovascular embolization of external carotid artery branches has gained significant attention as a treatment option for these cases [2, 11].

Currently, there is a paucity of single-center studies on endovascular embolization for PTH, with the majority of the literature consisting of case reports, most of which focus on pediatric cases [2, 3, 12, 13]. This study aims to evaluate the safety and efficacy of endovascular embolization in the treatment of post-tonsillectomy pseudoaneurysms in adults.

## Materials and methods

Ethical approval was obtained for this study, and the informed consent requirement was waived. The retrospective study was conducted on patients who underwent tonsillectomy in the Department of Otorhinolaryngology Head and Neck Surgery at the Second Affiliated Hospital of Xi'an Jiaotong University from January 2020 to July 2024. A total of 24 patients who experienced postoperative hemorrhage requiring rehospitalization were included in the analysis.

**Fig. 1:**
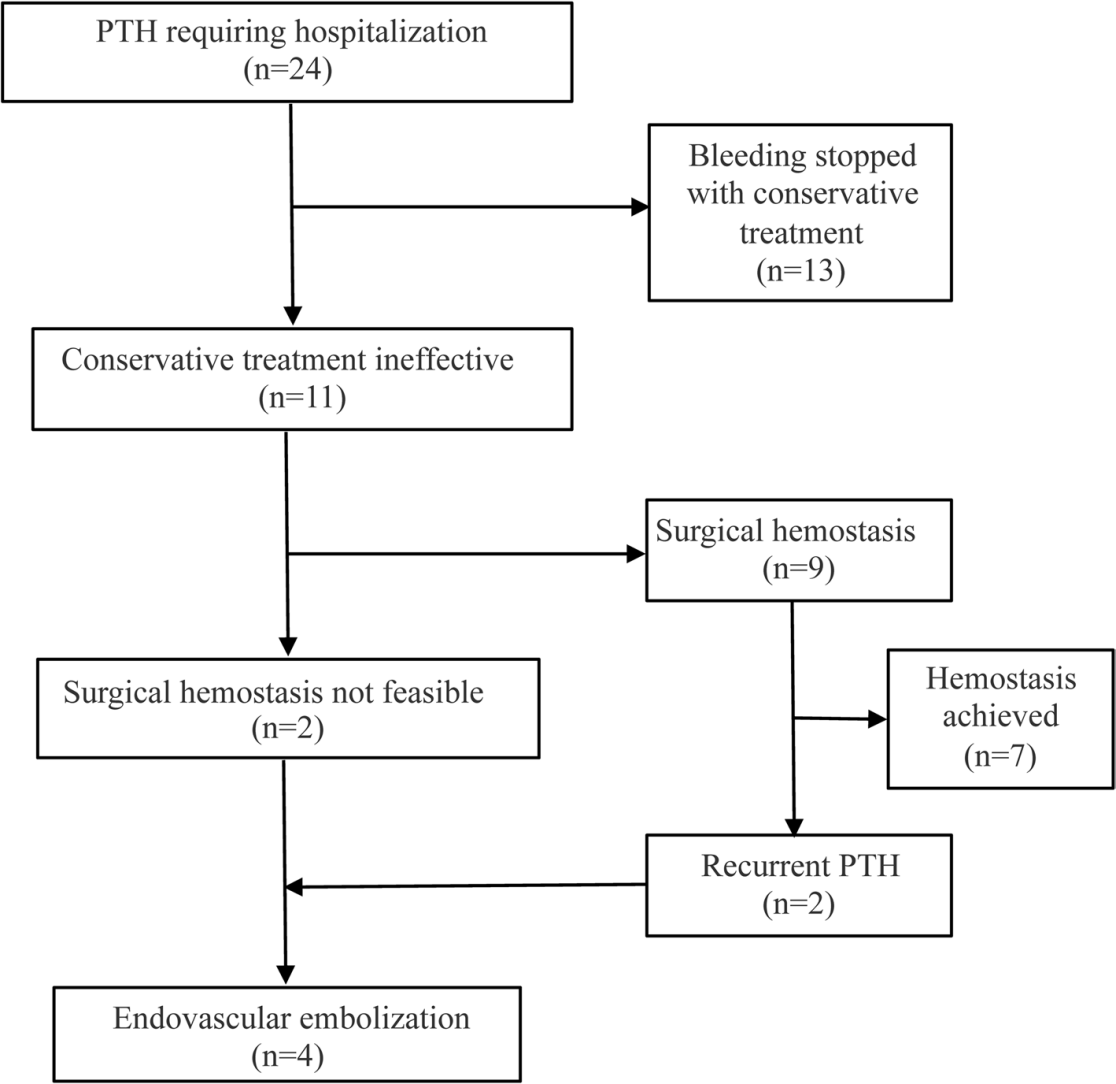
Flowchart of patients. PTH: post-tonsillectomy hemorrhage, N: number

Among these patients, 13 were successfully managed with conservative treatment—consisting of ice application, hydrogen peroxide gargles, hydrogen peroxide-soaked cotton ball compression, and hemostatic medications—without the need for further intervention. However, 11 patients required additional treatment because conservative treatment had proven ineffective. Of these, 9 patients underwent surgical hemostasis under general anesthesia, while 2 patients, who had no identifiable bleeding source upon laryngoscopy, received endovascular embolization at the interventional center. In the surgical hemostasis group, 7 patients achieved successful bleeding control, while 2 patients, who failed surgical hemostasis, subsequently underwent endovascular embolization. Ultimately, 4 patients were included in this study (Fig. 1). Additionally, we collected preoperative hemoglobin values of these patients before tonsillectomy and endovascular embolization. The difference between these values provided an approximate estimate of hemoglobin loss.

All patients underwent endovascular embolization with local anesthesia at the right femoral puncture site. In each case, a right femoral puncture was carried out, followed by the insertion of 5 French femoral sheaths (Terumo, Japan). Diagnostic digital subtraction angiography of both the external and internal carotid arteries was then performed using 5 French vertebral catheters (Cordis, USA). The presence of bleeding is directly evidenced by an arterial pseudoaneurysm or contrast agent extravasation, while arterial mural irregularities or abrupt truncation are indirect signs of bleeding.

All patients underwent endovascular embolization of the injured artery using superselective catheterization techniques. For larger arteries, embolization was performed with either coils alone or coils combined with n-butyl-2-cyanoacrylate (NBCA) glue (Glubran 2, GEM, Viareggio, Italy), depending on whether dangerous arterial connections were present at the injection site. For smaller arteries, the microcatheter was positioned as close as possible to the bleeding point, and NBCA glue was injected at a ratio of 1:2 with lipiodol. The objective of the injection was to occlude the segment of the parent artery containing the pseudoaneurysm with glue while preventing any distal leakage into normal branches or proximal backflow into the main external carotid artery. The coils used were IDC-18 interlocking detachable coils and pushable coils (Boston Scientific, USA).

## Results

Baseline characteristics of the included patients are shown in Table 1. 3 males and 1 female were included. In addition, two underwent tonsillectomy due to obstructive sleep apnea syndrome, one had tonsillectomy for recurrent tonsillitis and suppuration, and the other was treated with tonsillectomy for a tonsillar tumor. The bleeding started from 5 to 10 days after surgery. The number of bleeding episodes prior to undergoing endovascular embolization ranged from 3 to 7.

**Table 1:** Patients demographics and results

**NO.**	**Age** **(years)**	**Sex**	**Indications for tonsillectomy**	**Days of bleeding** **after surgery**	**Number of bleeding episodes**	**Hemoglobin Loss (g/L)**	**Injured artery**	**Embolic material**	**Recurrence of bleeding**	**Adverse events**
1	40	male	obstructive sleep apnea syndrome	5	7	55 (144/89)	Ascending palatine artery	NBCA	NO	NO
2	65	female	recurrent tonsillitis and suppuration	7	4	80 (127/47)	Main trunk of facial artery	Coil+NBCA	NO	NO
3	31	male	obstructive sleep apnea syndrome	10	5	77 (156/79)	Ascending palatine artery	Coil	NO	NO
4	42	male	tonsillar tumor	6	3	20 (106/86)	Lingual artery	NBCA	NO	NO

All patients underwent endovascular embolization only after conservative treatment or surgical hemostasis had failed and no further options were available. Specifically, Patient 1 had undergone multiple conservative treatments and one surgical hemostatic intervention; Patient 2 experienced one failed surgical hemostatic attempt and extensive gauze packing was also ineffective. Patients 3 and 4 had unsuccessful conservative treatment, with no active bleeding detected on laryngoscopy; thus, no surgical hemostasis was performed.

Four patients had varying degrees of hemoglobin loss, ranged from 20 to 80 g/L (Table 1). Patient 2 experienced a third episode of massive bleeding, leading to hypovolemic shock and cardiac arrest. Immediate cardiopulmonary resuscitation was performed by clinicians, successfully resuscitating the patient from the brink of death. Although endovascular embolization ultimately achieved hemostasis, the patient subsequently developed ischemic hypoxic encephalopathy and remains in the intensive care unit awaiting further hyperbaric oxygen therapy. According to the CIRSE classification system for complications’ reporting [14], patients 1, 3, and 4 were classified as grade 3b, while patient 2 was classified as grade 5.

**Fig. 2:**
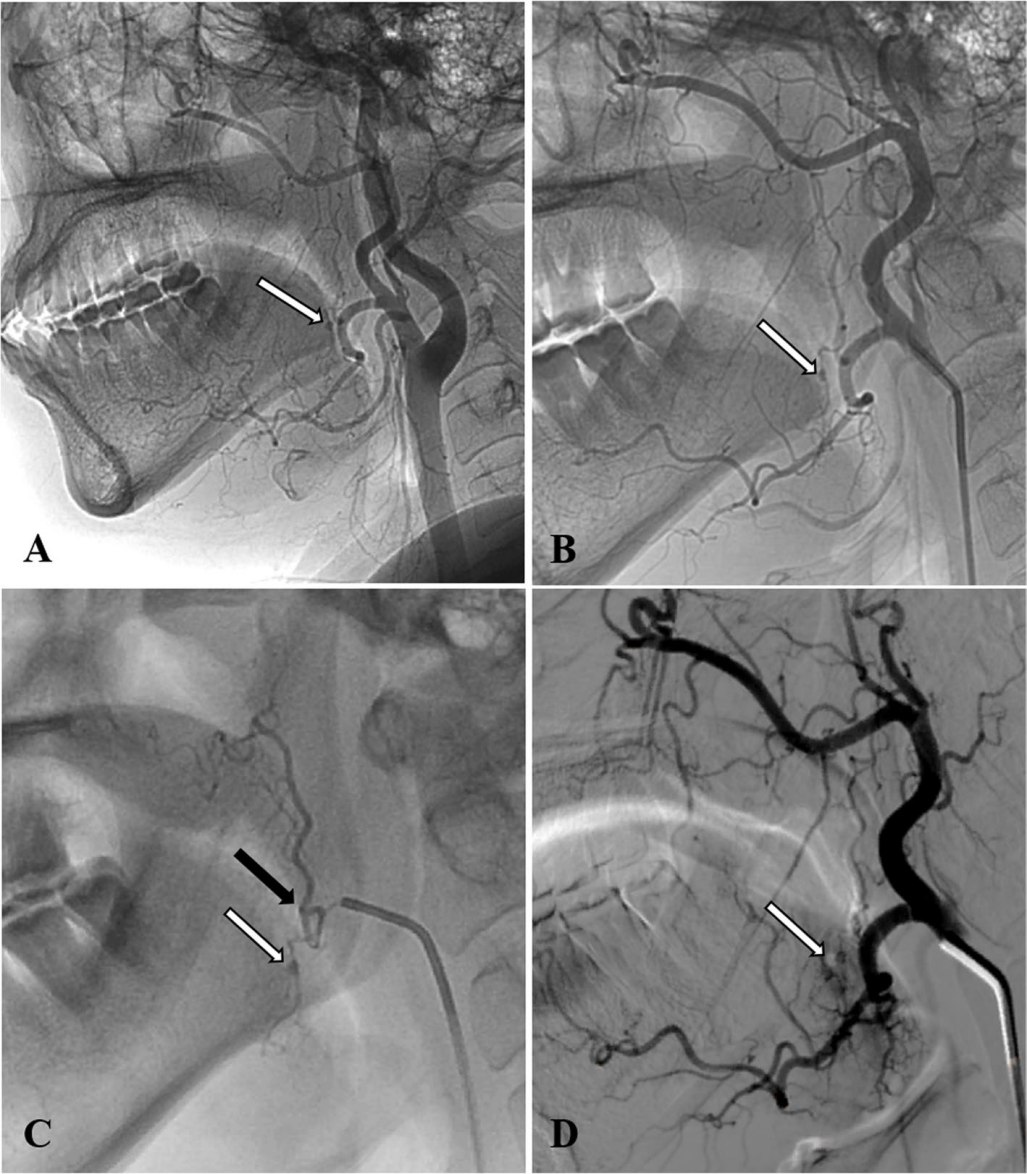
Pseudoaneurysm of the left ascending palatine artery tonsillar branch in Patient 1. **A** Lateral view of the left common carotid artery angiography reveals a very small pseudoaneurysm at the posterior border of the mandible (white arrow), **B** Subsequent selective catheterization of the external carotid artery revealed that this minute pseudoaneurysm (white arrow) originated from the first branch of the facial artery-the ascending palatine artery. **C** Further superselective angiography with a microcatheter into the ascending palatine artery confirmed that the pseudoaneurysm (white arrow) originated from the injured tonsillar branch of the ascending palatine artery. The black arrow indicates the tip of the microcatheter. Due to the small size of the tonsillar branch, we were unable to navigate into this tiny artery; therefore, NBCA glue was injected at the bifurcation. **D** Post-embolization left external carotid artery angiography showed that the pseudoaneurysm was successfully occluded by the glue (white arrow)

**Fig. 3:**
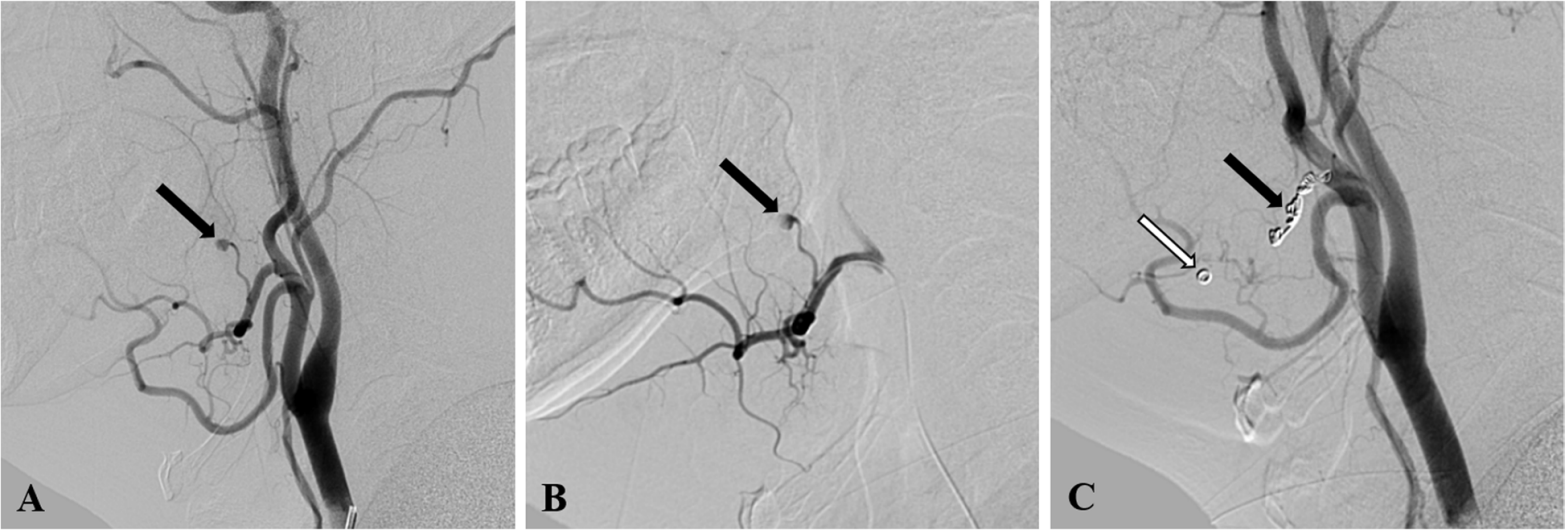
Pseudoaneurysm of the ascending palatine artery in Patient 3, treated by coiling. **A** Lateral view of the left common carotid artery angiography shows a pseudoaneurysm in the ascending palatine artery (black arrow). **B** Superselective catheterization of the left facial artery with a microcatheter confirmed that the pseudoaneurysm originated from the ascending palatine artery. Due to the unavailability of NBCA glue and concerns that the 2 mm diameter coil might not effectively deploy within the ascending palatine artery, we opted to advance the microcatheter to the distal segment of the facial artery beyond where it branches off into the ascending palatine artery. We then performed embolization of both the distal and proximal ends of the facial artery with the pushable coils to prevent recurrence of hemorrhage due to retrograde blood flow from the internal maxillary artery through collateral branches to the distal facial artery and subsequently supplying the ascending palatine artery. **C** Post-embolization angiography of the left common carotid artery (black arrow) shows successful occlusion of the pseudoaneurysm. Additionally, displacement of the coil towards the distal facial artery is noted (white arrow)

**Fig. 4:**
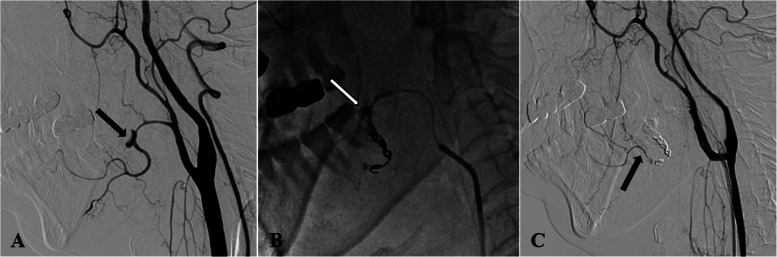
Management of a facial artery pseudoaneurysm in Patient 2 using the coil and NBCA Glue. A Lateral view of right common carotid artery angiography showing a pseudoaneurysm in the main trunk of the facial artery (black arrow). **B** A microcatheter was used to superselectively access the distal end of the pseudoaneurysm, where the interlocking detachable coils were deployed. NBCA glue was then injected into the proximal end of the pseudoaneurysm, achieving complete occlusion (white arrow). **C** Follow-up angiography of the common carotid artery confirmed successful occlusion of the pseudoaneurysm, with the distal facial artery well-visualized through collateral branches of the maxillary artery (black arrow)

**Fig. 5:**
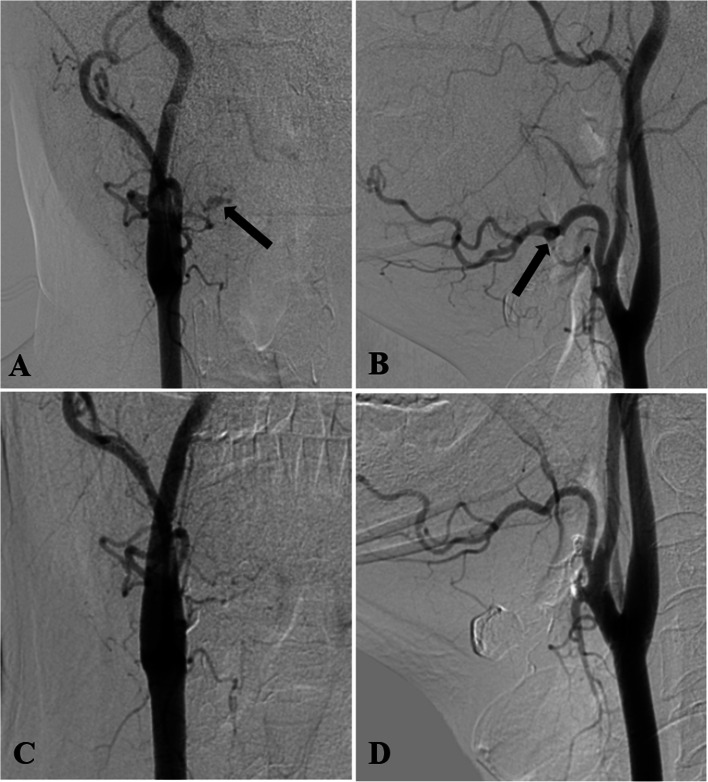
Treatment of a pseudoaneurysm of the lingual artery in Patient 4 using NBCA Glue. **A** Anteroposterior view of the right common carotid artery angiography reveals a pseudoaneurysm of the lingual artery (black arrow), clearly visible in the anteroposterior view. **B** Lateral view shows the pseudoaneurysm overlapping with the facial artery, making it less distinct. **C** Post-embolization, the anteroposterior view of the right common carotid artery angiography demonstrates successful occlusion of the pseudoaneurysm **D**. Lateral view post-embolization confirms that the pseudoaneurysm is no longer visible

The diagnostic angiogram revealed pseudoaneurysms involving the ascending palatine artery in two cases (Figs. 2 and 3), the facial artery main trunk in one case (Fig. 4), and the lingual artery in one case (Fig. 5). In our study, two patients were treated solely with NBCA glue, one patient was treated exclusively with coils, and one patient received embolization with a combination of coils and NBCA glue.

All four procedures were successfully completed, with effective occlusion of both the pseudoaneurysm and its feeding artery. The injection of NBCA glue was well-controlled, with no reflux into other significant proximal branches. Patient 1 presented with a pseudoaneurysm of the palatine tonsillar branch of the ascending palatine artery (Fig. 2). Because this branch was too narrow for the microcatheter to navigate, NBCA glue inevitably entered the palatine branch of the ascending palatine artery during embolization. However, the patient reported no discomfort postoperatively, and this was confirmed during a three-month follow-up. During the procedure for Patient 3, the first pushable coil experienced distal displacement after deployment (Fig. 3), which may have been attributed to either the relatively small diameter of the coil or the rapid blood flow. Nevertheless, this did not present any significant clinical concern. Following endovascular embolization, there were no subsequent episodes of bleeding, and no complications were reported in any of the cases.

## Discussion

Post-tonsillectomy pseudoaneurysm in adults can result in uncontrollable hemorrhage, potentially threatening life. Our study indicates that when surgical hemostasis or conservative treatments prove refractory, endovascular embolization presents an effective, feasible, and safe alternative option. NBCA glue may be considered the preferred embolic material, however, careful attention must be given to the critical techniques involved in its application.

Surgical hemostasis is the superior option in the treatment of PTH when conservative measures prove ineffective. However, research indicates that surgical hemostasis may sometimes be ineffective [1]. Endovascular embolization is a promising treatment option that offers several advantages. First, it provides both diagnostic imaging and therapeutic embolization. Second, superselective embolization enables precise occlusion of the bleeding vessel while preserving surrounding arteries. Third, it is minimally invasive, resulting in reduced trauma and faster recovery. Fourth, empirical embolization can be performed even with negative angiography results [1].

However, endovascular embolization for PTH has not yet received significant attention from otolaryngologists worldwide. In our study, Patient 1 underwent interventional embolization after experiencing five episodes of bleeding over seven days following surgical hemostasis. Patient 2 experienced cardiac arrest due to significant hemorrhage and underwent endovascular embolization only after four episodes of bleeding. Patient 3 experienced recurrent bleeding five times within two months post-tonsillectomy, resulting in three hospitalizations. Although all patients ultimately achieved hemostasis, the delay before the embolization procedure resulted in considerable blood volume loss, which had a substantial impact on the patients and necessitated a prolonged period for adjustment and recovery.

Pseudoaneurysms are particularly unstable and can lead to sudden, uncontrollable massive hemorrhage due to various factors. In this study, all patients with angiography-confirmed pseudoaneurysms exhibited this characteristic pattern of bleeding. Massive hemorrhage caused by pseudoaneurysms of the pharyngeal artery is an exceedingly rare and severe complication in patients undergoing tonsillectomy [15]. To date, there have been no reports of any examinations other than angiography that can confirm this condition. The operator must meticulously analyze the angiographic images, paying close attention to the course and morphology of all feeding arteries to the tonsils.

The most effective embolization strategy for a pseudoaneurysm is to achieve permanent occlusion at both the distal and proximal ends of the parent artery. Utilization of a soft-tipped microguidewire is essential for safe microcatheter navigation to the pseudoaneurysm's distal end, as improper technique may rupture its fragile wall, causing severe hemorrhage.

Different embolic materials have been described for embolization of post-tonsillectomy pseudoaneurysm, including Polyvinyl alcohol (PVA) particles, gelatin sponges, coils, and NBCA glue. Previous studies have indicated that coils are among the optimal embolic agents [16, 17]. However, when the bleeding artery is too small to enable the use of coils of appropriate size, liquid embolic materials such as NBCA glue become the only reliable embolic option, it can diffuse into even the smallest vessels, achieving permanent occlusion of both the pseudoaneurysm and the parent artery. Patient 1 serves as a compelling example (Fig. 2). In fact, NBCA glue is applicable for larger vessel ruptures. It achieves reliable hemostasis while reducing costs, offering advantages that coils alone cannot provide, as demonstrated in patient 2 (Fig. 4). Moreover, to our knowledge, there are currently no reports on the use of NBCA glue for the treatment of pseudoaneurysms of the ascending palatine artery, nor on the combined use of NBCA glue and coils for treating facial artery pseudoaneurysms.

The decision to use glue as an embolizing material for the post-tonsillectomy pseudoaneurysm requires careful consideration of the following points: Firstly, for large parent arteries of the pseudoaneurysm with high blood flow velocity, coils should first be deployed distally, followed by NBCA glue injection proximally. This reduces blood flow, promoting glue polymerization and solidification, while protecting distal branches from infiltration (Fig. 4). Secondly, once the glue fills the pseudoaneurysm and refluxes proximally, stop the injection. If the glue reaches the microcatheter tip, wait about one minute before withdrawing to allow solidification and secure bonding to the vessel wall. This prevents displacement of the glue to other branches of the external carotid artery or the internal carotid artery during microcatheter removal. Thirdly, the glue should not be overly diluted, as this can extend its coagulation time, increasing the risk of it being carried by blood flow into the peripheral capillary beds and causing tissue necrosis. Additionally, great care should be taken with arteries that may have anastomoses with intracranial arterial branches, such as the ascending pharyngeal artery, the middle meningeal artery originating from the internal maxillary artery, and the angular artery, etc.

This study has several limitations. The first limitation is the small patient sample. Because the conservative measures and surgical hemostasis are the superior option in the treatment of PTH, small number of patients can be included in our study. Another limitation was no control group utilizing other embolic materials for comparison. Finally, this is a retrospective study, and further prospective study is needed to investigate the safety and efficiency of endovascular embolization to treat PTH.

## Conclusion

Endovascular embolization is an effective, feasible, and safe treatment option for refractory hemorrhage resulting from post-tonsillectomy pseudoaneurysm in adults. NBCA glue can be an effective and appropriate embolic material, but attention must be paid to the critical techniques involved.

## Data Availability

Data available on request from the authors.
